# Hospital Construction Cost Affecting Their Lifecycle: An Italian Overview

**DOI:** 10.3390/healthcare9070888

**Published:** 2021-07-14

**Authors:** Leopoldo Sdino, Andrea Brambilla, Marta Dell’Ovo, Benedetta Sdino, Stefano Capolongo

**Affiliations:** 1 Department Architecture Built Environment Construction Engineering (DABC), Politecnico di Milano, via G. Ponzio, 31, 20133 Milan, Italy; leopoldo.sdino@polimi.it (L.S.); andrea1.brambilla@polimi.it (A.B.); stefano.capolongo@polimi.it (S.C.); 2 Department of Architecture and Urban Studies (DAStU), Politecnico di Milano, via E. Bonardi, 3, 20133 Milan, Italy; marta.dellovo@polimi.it

**Keywords:** construction cost, hospitals, Italy, healthcare, estimation tool, questionnaire

## Abstract

The need for 24/7 operation, and the increasing requests of high-quality healthcare services contribute to framing healthcare facilities as a complex topic, also due to the changing and challenging environment and huge impact on the community. Due to its complexity, it is difficult to properly estimate the construction cost in a preliminary phase where easy-to-use parameters are often necessary. Therefore, this paper aims to provide an overview of the issue with reference to the Italian context and proposes an estimation framework for analyzing hospital facilities’ construction cost. First, contributions from literature reviews and 14 case studies were analyzed to identify specific cost components. Then, a questionnaire was administered to construction companies and experts in the field to obtain data coming from practical and real cases. The results obtained from all of the contributions are an overview of the construction cost components. Starting from the data collected and analyzed, a preliminary estimation tool is proposed to identify the minimum and maximum variation in the cost when programming the construction of a hospital, starting from the feasibility phase or the early design stage. The framework involves different factors, such as the number of beds, complexity, typology, localization, technology degree and the type of maintenance and management techniques. This study explores the several elements that compose the cost of a hospital facility and highlights future developments including maintenance and management costs during hospital facilities’ lifecycle.

## 1. Introduction

### 1.1. Healthcare System and Facilities

According to the World Health Organization (WHO), a health system involves all organizations, people and actions that have the promotion, restoration and maintenance of health status as their primary objective [1]. The health system is a very important component of the overall state activities, representing a considerable amount of the total annual expenditures, reaching up to 9% to 15%, respectively, in Italy’s and the United States’ (USA) gross domestic product (GDP) [2]. Within healthcare systems, hospitals play a fundamental role. They have a great impact on the GDP and job creation, and they account for a substantial proportion of the healthcare budget: about 50% in many Western European countries, and 70% or more in Eastern European countries [3]. Although they are commonly intended as “an institution which provides beds, meals, and constant nursing care for its patients while they undergo medical therapy at the hands of professional physicians […] striving to restore its patients to health” [4], today, according to the WHO, hospitals are instrumental for care coordination and integration and have a key role to play in supporting other healthcare providers (including primary healthcare) and in community outreach and home-based services. They also often provide a setting for education of doctors, nurses and other healthcare professionals and are a critical base for clinical research. They must be resilient and able to maintain and scale up services in emergency situations [5].

### 1.2. The Problem of Hospital Building Construction Cost

Scholars agree on conceiving hospitals as complex organizations, and not simple entities, which are subjected to both external and internal levers of change and are fundamental for the entire population of a country; it is estimated that due to these changing needs, about 50–60 years is the optimal lifecycle of a modern healthcare building [6]. Given the continually changing nature of the health sector, this requires consistent and continuous investments into new and updated facilities and equipment. Such investments offer scope to policymakers to shape hospital performance through strategic and financial decisions, although the precise opportunities depend on the ownership, funding and regulatory systems within which the specific facility falls [7]. Additionally, healthcare is always seen more by developers and investors as a valuable alternative asset class, with clinics, nursing homes and assisted homecare, which are reported among the European and Italian top trends in real estate investments. The investments required in building or renovating a hospital are usually so large that the organization must base that investment, along with its programming and design decisions, on rigorous analysis of long-term consequences. The scientific and technical communities agree on estimating the initial cost to be equivalent to about one year of running costs [8]. Since a well-designed and well-built hospital can contribute to improving quality and medical outcomes, reducing expenses and improving revenues, it is therefore clear that wrong decisions in the design and planning phase might considerably impact the operation phase and its running costs [9,10].

### 1.3. State of the Art of Healthcare Built Assets and Urgent Needs: The Case of Italy

As it has been already stated, hospitals, given their changing and challenging environment, can be considered as complex and risky assets to deal with, and several difficulties emerge when trying to estimate the construction cost of such assets in order to evaluate the feasibility of healthcare operations. In Italy, the regionalization and the health expenditure containment need, due to the rationalization action over the last 20 years, has led to an exponential decrease in the number of hospitals in the whole territory; furthermore, compared to 1997, the overall total beds have decreased by 39% [11]. The Italian hospital asset is considered advanced and well functioning, and the whole organizational system is also positively judged at international levels, but most hospitals are old, unorganized, dilapidated and sometimes dangerous [12]. Obsolescence is the biggest issue; indeed, 60% of the overall facilities were constructed more than 60 years ago [13,14]. Around 70% of public hospitals in the south and in the center of Italy were constructed before 1970, with this value reducing to 54% in the northeast, and to 65% in the northwest of the country [12]. An indication to evaluate Italian hospitals’ infrastructure state can be furnished by the hospitals’ construction year. This indicator allows drawing important information about hospital facilities’ age, whose construction may have been opened 10 or 20 years before the start-up of the hospital itself. A hospital structure’s age is particularly critical since it makes the hospital poorly adaptable to technologies’ and sanitary techniques’ rapid evolution and nonfunctional to the patients’ changing needs, and flexibility is quite an important characteristic for hospitals, given their constantly changing environment [6]. Nowadays, the matter is to understand and evaluate whether the existing healthcare assets should be refurbished, renovated or constructed again when they reach the end of their lifecycle. The poor replacement which occurred in the last 20 years entailed massive investments in refurbishments, and such renovations limited the issues deriving from the structures’ age, but they did not solve the problem at all. Therefore, the structures remain inadequate for the provision of modern medical assistance. It is evident that during the worldwide COVID-19 pandemic, all national health systems and structures have been stressed in order to cope with the huge assistance request by the population [15]; therefore, new investment programs are arising such as the Next Generation EU in Italy [16], or the UK Health Infrastructure Plan [17], for building several new facilities. Nevertheless, although there is a significant number of expert practitioners in hospital design, there are still missing reliable and easy-to-use instruments to parametrize the cost of healthcare facilities to make reasonable plans and investments for increasing the quality of the overall health system with realistic and coherent amounts of money.

### 1.4. Study Objective and Paper Structure

Therefore, this research analyzed, first of all, different sources in order to determine the overall expenditure needs when constructing a new hospital. This contribution is the first step to systemizing a set of data which is quite difficult to collect and elaborate. Moreover, by distinguishing the different construction cost outputs, a comparative analysis between them could be carried out, reasoning about the disparities between the sources. Furthermore, the research aims, after determining hospitals’ construction costs, to define and categorize a series of parameters which influence the construction cost definition the most.

In detail, this paper is structured in five sections. The first section frames the problem of healthcare buildings’ construction cost, with reference to Italy; the second section presents the methodological framework adopted in the analysis, and its application is presented in the third section, divided into theoretical, practical and interactive phases. The fourth section reviews the results obtained and develops a preliminary estimation tool in order to understand which factors impact hospitals’ construction costs the most, and the last section sums up the conclusions and analyzes the possible further implementation of this research.

## 2. Materials and Methods

**Figure 1:**
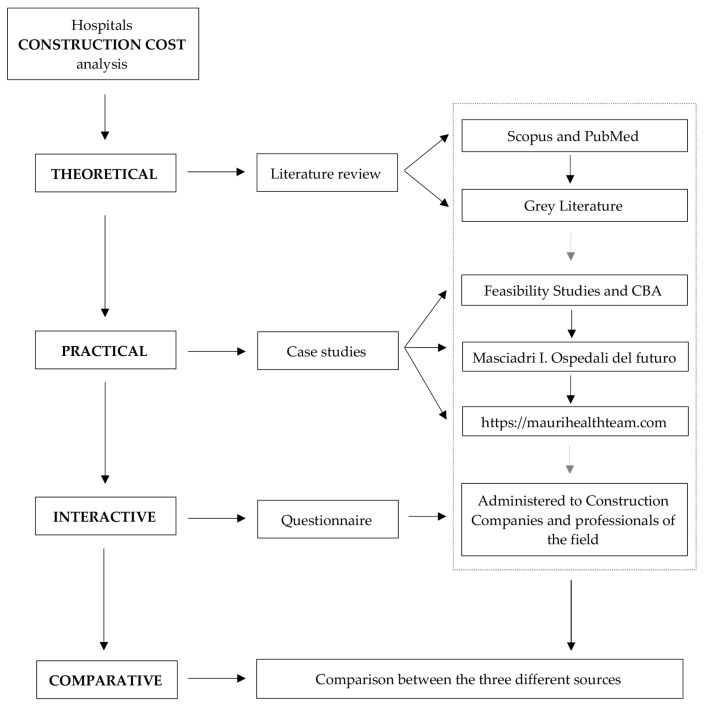
Methodological approach flowchart with the identification of the four phases.

This paper proposes different contributions to deal with such a complex topic. Indeed, the methodological approach followed can be divided into four different phases: (i) theoretical, (ii) practical, (iii) interactive and (iv) comparative. The meaning of all four phases is to find data and information on the construction cost of Italian hospitals through different sources in order to have independent groups of data to compare and discuss. During the first phase, (i) theoretical, the scientific literature and the gray literature were analyzed in order to understand how other scholars face the problem; indeed, reports, articles and contributions were examined. Then, the second phase, (ii) practical, saw the analysis of Italian case studies taken from the available feasibility studies and constructed or under-construction hospital cases. The third phase, (iii) interactive, involved a panel of experts, working in a hospital, replying to an ad hoc designed questionnaire with a specific inquiry into the hospital construction cost. In the last phase, (iv) comparative, a comparison between the three different sources was conducted. The four phases are represented in Figure 1.

### 2.1. Theoretical Phase: Literature Review

The first step examined whether there were contributions on the topic of hospital construction costs. Through the Scopus and PubMed databases, an analysis was carried out in the period May–June 2020 by using a set of keywords.

The first keywords used in Scopus were “hospital” and “construction cost”, limited to the field of business, management and accounting, returning a result of 74 papers; by reading the title, 47 of them were excluded since they were not relevant considering this research’s purpose. Then, between the 27 articles that remained, 13 were excluded by reading the abstract. Six of the remaining fourteen papers were analyzed by author(s), nationality, year, case study and its application country, methodology used, cost definition and value. However, it was possible to define a cost definition only for three of them, and the construction cost value for one of them, but it was expressed in an overall general way.

Then, while handling the topic in a more general way, the keywords inserted in the database were “hospital” and “cost”, with the same limitation as above, generating 2.969 results; therefore, they were processed for the last 5 years, returning 752 results, concentrated on clinical studies on costs, and not the hospital as an asset.

The last set of keywords used contained “construction cost” and “healthcare”, with the same limitation as above, generating no results. Additionally, words synonymous with the word hospital were used to make the research more effective, such as “healthcare facility”, “healthcare environment”, “hospital building” and “health building”. They generated some results, but none of them were useful for this research’s purpose.

Moving to the PubMed database, the same keywords were used; however, most of the results focused on the medicine technique and hospitalization cost fields.

The main output of this phase was that the subject is discussed in the literature in a partial way, meaning it was not possible to define, in this first part of this research, the value of hospitals’ construction costs. Furthermore, it was decided to continue with the gray literature analysis.

The second step was to analyze the available gray literature on the internet by using the keywords “hospital”, “construction cost”, “feasibility study” and “Italy”.

### 2.2. Practical Phase: Case Studies Analysis

**Figure 2:**
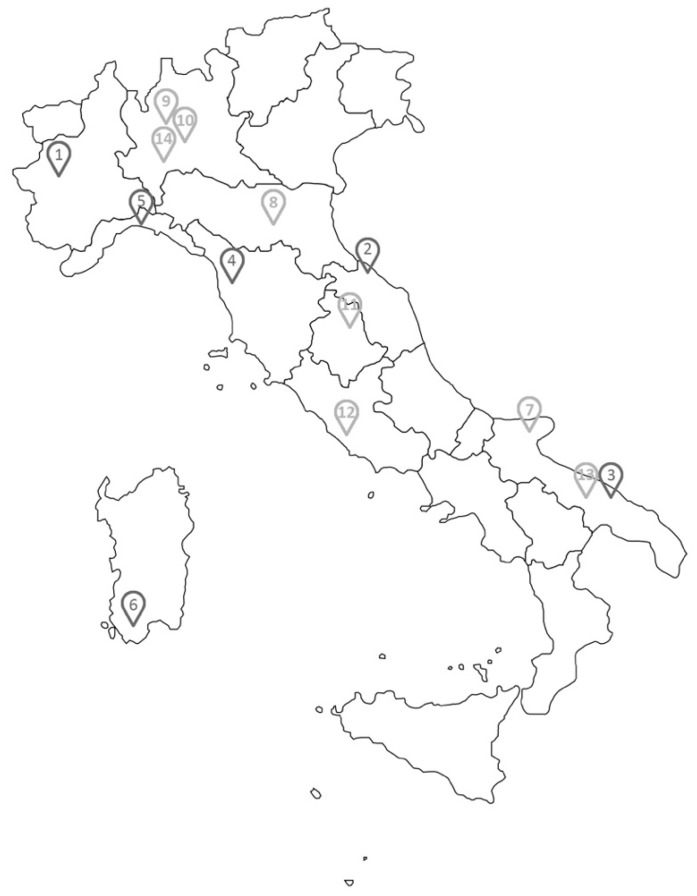
Case studies’ localization.

Data were collected regarding Italian hospitals’ construction cost through available case studies, by analyzing both their feasibility studies and, in some cases, cost–benefit analysis and constructed hospital cases. The localization of the 14 hospital case studies analyzed can be seen in Figure 2.

The sources for this paragraph were various. Some of them came from feasibility studies found on the net by searching the following keywords: “feasibility study”, “new hospitals construction”, “cost-benefit analysis” and “Italy”; additionally, a technical book [18] and a website gathering hospitals under construction [19] were exploited. An overview of the healthcare structures considered in the analysis can be found in Table 1.

### 2.3. Interactive Phase: Questionnarie Submission

The third step was developed by administering a questionnaire to construction companies and experts of the real estate sector. This expert panel was selected according to the research objective, involving medium–large-sized hospital facilities’ technical directors.

The questionnaire was divided into six sections as follows: the first one contained general information about the company or the person who was filling out the questionnaire; the second section asked for the construction cost of the infrastructure in the hospital; the third section asked for the overall construction cost of a hospital divided into the three different complexity typologies (HUB, SPOKE and BASE—the three hospital complexity typologies are differentiated from one another according to the different intensities of care that a hospital provides) and differentiated between the cost in EUR/sqm and EUR/bed; the fourth section regarded the building box pluri-parametric costs in EUR/sqm, asking for the construction cost of the different areas inside a hospital; the fifth section required indicating a value in EUR/sqm for the technological units; and the last section asked for the furniture cost. For each voice, a reference value taken from the literature was indicated. The full list of questions can be found in Appendix A.

### 2.4. Comparison Phase

The last step of this research was to draw a comparative analysis of the sources analyzed in this research, not only to develop some considerations and thoughts about the overall construction cost of hospitals but also to understand how the value is going to change according to the different sources and the possible reasons of such output.

## 3. Results

### 3.1. Literature Review

As it was mentioned in the methodology section, the search in scientific databases did not return any significant output; therefore, the gray literature was analyzed, and the main results are reported below.

The first contribution analyzed was written by IRES Piedmont, which is a Piedmont region research institution organized by Regional Law 43/91, and which publishes a report each year about regional socio-economic and territorial trends and carries out analysis, both of scenarios and circumstances, of Piedmont’s major socio-economic and territorial phenomena. Indeed, in 2018, it published a report which analyzed the topic of “Hospitals. Theoretical construction and management costs” [26].

The report examines the construction cost per square meter and the construction cost per bed, dividing them into the three different hospital intensity categories (high, medium and low). Additionally, two online sources were added in the reference table. A summary of the small number of contributions found through the literature investigation is reported in Table 2.

### 3.2. Case Study Analysis

It should be underlined that the construction cost taken into consideration in all the case studies is the construction cost only (the one which usually represents 75% of the total cost), excluding the area acquisition, the urban expenses, the furniture, the technological nods, etc. (which are often indicated as 25% of the overall construction cost), the reasoning following the Public Works Price List guidelines. This choice was also made in order to be coherent when comparing the literature with the case studies [29].

The output of this part of the analysis can be seen in Table 3, where the results are reported. There is a differentiation between two different results: the first one is the computation of the construction cost indicated in euros per square meters, while the second result is the computation of the construction cost in euros per bed, which can be found inside a single hospital.

### 3.3. Questionnaire

The main output of this part of the analysis regards the fourth section of the questionnaire, the building box mono-parametric construction costs; indeed, in Table 4, the results of the values combining the different answers can be seen. The questionnaire was sent to 25 people, with 7 replies received (see Appendix A).

## 4. Comparison of the Results Achieved through Different Sources

A comparison between the different sources exploited is provided and further commented on, starting from Table 5.

A differentiation was made between the feasibility studies and case studies, with the former gathering studies conducted in the preliminary phase of a project, and the latter considering cases of already constructed hospitals.

**Figure 3:**
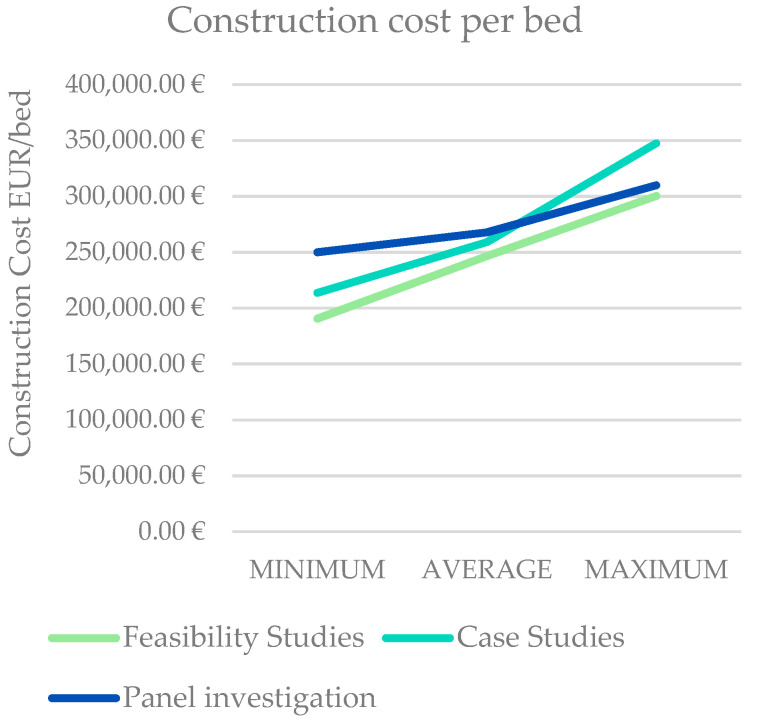
Comparison between construction cost per square meter (EUR/sqm) and per bed (EUR/bed) expressed in feasibility studies (literature), case studies and panel investigation.

The main consideration, considered between the conclusion value (average between feasibility studies, case studies and panel investigation) and the gray literature value, is that the cost per square meter is a bit overestimated in the gray literature (IRES contribution) [26]. The maximum overestimation occurs in the maximum cost, with a difference of EUR 332.5 (15.3%), and the average value differs by EUR 236.53 (12.7%), and the minimum value by EUR 201.91 (11.2%) (Figure 3).

On the other hand, when analyzing the cost per bed values, it should be underlined how the values are consistent between them. The lowest average value is in the feasibility studies, while the conclusion value (average of feasibility studies, case studies and panel investigation) differs from the gray literature average value of EUR 5779.87, corresponding to 2.3%.

A different reasoning should be considered for the minimum and maximum values.

In the case of the minimum value, the highest value in EUR/bed was computed by the panel, with EUR 250,000, and the feasibility studies computed the lowest value of EUR 190,605.35; the more consistent values are the gray literature and case studies values of, respectively, EUR 216,000.00 and EUR 213,618.18.

In the case of the maximum values, the gap between the minimum and the maximum is EUR 47,504.46. The maximum value provided is in the case studies, EUR 347,504.46, while the other three are quite consistent, from EUR 300,000 to EUR 310,000, corresponding to a 15.8% variation.

Please note that the three values (average, minimum and maximum) are tied to the degree of complexity of the hospitals classified as a HUB, SPOKE or BASE hospital [26]; the differences between the values do not overcome 10%; therefore, such gap should be attributed to the different structures’ degree of complexity.

The second output is a preliminary estimation tool in order to identify the minimum and maximum variation in the cost when programming the construction of a hospital. Such tool could be very useful in the early stages of a project, meaning the feasibility phase or the early design stage. It might be used by all those figures which have to deal with such phases: decision makers, town planners, designers, etc.

This tool was constructed by identifying some key characteristics of hospital facilities: complexity, beds, typology, localization, technology and age.

In order to utilize this tool, following the analysis conducted, the “hospital type”, according to the characteristics mentioned above, was identified. This means that the average attribute for each characteristic was identified:

- For the complexity, the average-intensity facilities are SPOKE hospitals;
- The average beds, according to the analysis carried out in the case studies, total 450;
- The typology identified in the average attributes is the poly-block;
- The localization, whose construction cost is more similar to the Italian average (an ideal value which corresponds to 0), is Calabria;
- The technological capital state is the medium one;
- The average age of a hospital facility is indicated as 30 years.

This application, which considers the average characteristics of the hospital type as an applicative example, is useful to determine how the cost can vary depending on whether some of the characteristics change, as shown in Table 6. The computation of the percentages was conducted according to practical experience in the field and a dialogue with the National Construction Association (ANCE).

Maintenance and management have the highest weight in the operation, followed by the localization, the complexity (meaning HUB, SPOKE or BASE hospital), typology, technology and the number of beds [30].

The reference is the hospital type already described, and if the characteristics of the hospital change, the values change. Indeed, if the hospital is more complex compared to the hospital type described, the values in percentage increase; on the other hand, if the hospital is less complex compared to the hospital types, the values in percentage decrease. It is a subjective assessment which needs to be verified by an expert based on the characteristics of each hospital.

The percentages reported above are part of an applicable example of a hospital’s average data; therefore, they are subject to changes by the evaluator when in the presence of different hospital characteristics.

## 5. Discussion and Implications of Findings

This contribution considered the construction cost in hospital facilities from different contributions and different perspectives, the literature, some case studies and a discussion with experts in the field. It was fundamental to compare the different sources’ data and understand whether there was a variation in the cost component, and, if so, the reasons behind it. The impact that such tool might have when designing a new hospital or deciding whether to construct a new hospital or not is quite huge. First of all, only by developing this type of analysis can the categories that impact the construction cost component the most be understood. Additionally, it demonstrates how the construction cost and the maintenance and management cost are tied up, given the fact that the more years the hospital has, the more the maintenance and operating activities are going to impact on the facility [31]. Finally, given this evidence, it might be more convenient to construct a new hospital, rather than continue the maintenance and management operations over its lifecycle, which is indicated to be 60 years.

The further step taken with the preliminary estimation tool of the characteristics which influence the overall hospital construction cost underlined that the maintenance and management component is the one that weighs the most in the overall cost. This tool could be applied in all of the different situations when deciding on the new construction of a hospital facility, which, as defined before, is more convenient than intervening in an asset with massive refurbishment or partial reconstructions.

This tool is quite useful in the feasibility phase or the preliminary design of a hospital facility; indeed, it could be used by decision makers, stakeholders and all the figures involved in hospitals’ construction evaluation process from the very early stages.

## 6. Conclusions and Limitations of the Study

This research was approached following three different paths: a scientific literature review, case study analysis and panel investigation through a questionnaire. One of its limitations is, first of all, the number of case studies considered; indeed, it was decided to focus the spectrum amplitude on Italian examples without comparing them to some international cost variances. Therefore, a broader and more international panel might provide additional references, even if the cost in each country might be influenced by different external drivers.

A final consideration concerns the lifecycle of hospital facilities. The construction cost is indicated in the literature as a minimum part of the asset’s overall lifecycle cost, which is composed of the initial cost, such as the investment evaluation, the design and the construction; then the running cost, maintenance cost and use cost; and, finally, the closing cost, which comprehends the refurbishments, dismantling and sale. Indeed, as the overall lifecycle of hospital assets is indicated in the literature as 60 years, great attention should be paid to the running phase of the facility, particularly the maintenance and operating costs, which, for a hospital, are critical, given the special nature of the assets and their importance for the whole community.

Under this perspective, and starting from the limitations of the study, such as the number of cases and the limited amount of data on operating costs, further developments might address the relationship between the construction cost and the maintenance and management cost applied to hospitals’ overall lifecycle. Moreover, the development of a phase for the validation of the results obtained, together with a panel of selected experts and construction companies, would be interesting.

## Figures and Tables

**Table 1:** Analyzed case studies overview.

N.	Region	City	Source	Hospital Case Study
1	Piedmont	Moncalieri	Feasibility Study (2018) [20] *	Nuovo Ospedale Unico dell’Azienda Sanitaria Locale TO5
2	Marche	Pesaro	Feasibility Study (2014) [21] *	Nuovo Ospedale Marche Nord
3	Apulia	Monopoli-Fasano	Feasibility Study and CBA (2013) [22] *	Ospedale del Sud Est Barese
4	Tuscany	Lucca	Preliminary Feasibility Study (2011) [23] *	Ospedale Unico della Valle del Serchio
5	Liguria	Genoa	Feasibility Study (2010) [24] *	Ospedale Galliera
6	Sardinia	San Gavino	Feasibility Study (2010) [25] *	Nuovo Ospedale di San Gavino
7	Lombardy	Brescia	(ongoing) [19]	Fondazione Poliambulanza
8	Emilia-Romagna	Bologna	(2016) [18]	Ospedale S. Orsola Malpighi
9	Lombardy	Monza Brianza	(2011) [18]	Ospedale di Vimercate
10	Lombardy	Milan	(2010) [18,19]	Ospedale di Legnano
11	Umbria	Perugia	(2008) [18,19]	Ospedale di Gubbio e Gualdo Tadino
12	Lazio	Roma	(2008) [18]	Policlinico Universitario Campus Bio-medico
13	Apulia	Bari	(2006) [18]	Ospedale Miulli
14	Lombardy	Rozzano	(2005) [19]	Istituto Clinico Humanitas

* Note that the year indicated in the source column refers to the feasibility studies’ publishing date, while for the other two references, the date refers to the end of hospital construction works.

**Table 2:** Hospital construction cost in EUR/square meter according to gray literature.

Hospital Type	Unit Costs by Square Meter (EUR/Sqm) Unit Costs by Beds (EUR/Bed)		
	**Minimum**	**Reference**	**Maximum**
High complexity	2000 EUR/sqm 240,000 EUR/bed	2200 EUR/sqm 264,000 EUR/bed	2500 EUR/sqm 300,000 EUR/sqm
Medium complexity	1900 EUR/sqm 228,000 EUR/bed	2100 EUR/sqm 252,000 EUR/bed	2400 EUR/sqm 288,000 EUR/bed
Low complexity	1800 EUR/sqm 216,000 EUR/bed	2000 EUR/sqm 240,000 EUR/bed	2300 EUR/sqm 276,000 EUR/bed
Hospital 1 [27]	1900 EUR/sqm 292,000 EUR/bed		
Hospital 2 [28]	2040 EUR/sqm N.A. EUR/bed		

**Table 3:** Case studies’ construction cost per square meter and bed.

N.	Region	City	Hospital	EUR/Sqm	EUR/Bed
1	Piedmont	Moncalieri	Nuovo Ospedale Unico dell’Azienda Sanitaria Locale TO5 Nuovo Ospedale Marche Nord	EUR 1747.4	EUR 241,651.27
2	Marche	Pesaro	Ospedale del Sud Est Barese Ospedale Unico della Valle del Serchio	EUR 1658.18	EUR 229,541.53
3	Apulia	Monopoli-Fasano	Ospedale Galliera Nuovo Ospedale di San Gavino	EUR 1588.38	EUR 190,605.35
4	Tuscany	Lucca	Fondazione Poliambulanza Ospedale S. Orsola Malpighi	EUR 2145.60	EUR 300,384.00
5	Liguria	Genova	Ospedale di Vimercate Ospedale di Legnano	EUR 1805.51	EUR 272,669.39
6	Sardinia	San Gavino	Ospedale di Gubbio e Gualdo Tadino Policlinico Universitario Campus Bio-medico	EUR 1400.97	EUR 243,761.95
7	Apulia	S. Giovanni Rotondo	Ospedale Miulli	EUR 2090.91	EUR 230,000.00
8	Emilia Romagna	Bologna	Nuovo Ospedale Unico dell’Azienda Sanitaria Locale TO5 Nuovo Ospedale Marche Nord	EUR 1903.21	EUR 265,097.90
9	Lombardy	Vimercate	Ospedale del Sud Est Barese Ospedale Unico della Valle del Serchio	EUR 1873.38	EUR 295,222.80
10	Lombardy	Milano	Ospedale Galliera Nuovo Ospedale di San Gavino	EUR 2166.54	EUR 275,740.88
11	Umbria	Perugia	Fondazione Poliambulanza Ospedale S. Orsola Malpighi	EUR 1696.15	EUR 213,681.18
12	Lazio	Roma	Ospedale di Vimercate Ospedale di Legnano	EUR 2391.28	EUR 347,504.46
13	Apulia	Bari	Ospedale di Gubbio e Gualdo Tadino Policlinico Universitario Campus Bio-medico	EUR 1932.42	EUR 220,449.11
14	Lombardy	Rozzano	Ospedale Miulli	EUR 2211.13	EUR 224,678.83

**Table 4:** Panel investigation construction cost output.

	Cost (EUR/Sqm)			Cost (EUR/Bed)	
**Average**	**Minimum**	**Maximum**	**Average**	**Minimum**	**Maximum**
EUR 1863.7	EUR 1598.09	EUR 2167.85	EUR 257,779.87	EUR 218,095.51	EUR 319,296.15

**Table 5:** Construction cost comparative analysis.

Contribution	Cost (EUR/Sqm)			Cost (EUR/Bed)		
	**Average**	**Minimum**	**Maximum**	**Average**	**Minimum**	**Maximum**
Gray Literature	EUR 2100.00	EUR 1800.00	EUR 2500.00	EUR 252,000.00	EUR 216,000.00	EUR 300,000.00
Feasibility Studies	EUR 1724.43	EUR 1400.97	EUR 2145.60	EUR 246,435.58	EUR 190,605.35	EUR 300,384.00
Case Studies	EUR 2033.13	EUR 1696.15	EUR 2391.28	EUR 259,046.90	EUR 213,681.18	EUR 347,504.46
Panel Investigation	EUR 1832.86	EUR 1697.14	EUR 1966.67	EUR 267,857.14	EUR 250,000.00	EUR 310,000.00
Conclusion	EUR 1863.47	EUR 1598.09	EUR 2167.85	EUR 257,779.87	EUR 218,095.51	EUR 319,296.15

**Table 6:** Construction cost variation preliminary estimation tool.

Factor	Minimum	Medium	Maximum
Complexity	−9%	SPOKE	9%
Beds	3%	450	−3%
Typology	−6%	Poly-block	6%
Localization	−12%	Calabria	12%
Technology	−5%	Medium	5%
Maintenance and Management	−36%	30	45%

## Data Availability

Not applicable.

## Appendix A. Full Questionnaire Addressed to the Expert Panel

**Table A1:** Questionnaire.

Name of the Company
Did the Company participated to hospitals construction in the last 10 years?
Would you like the questionnaire results to be anonymous?

**Table A2:** Infrastructure costs.

In this section, it is required to insert a construction value in EUR/lm or EUR/sqm for each indicated category, according to own experience in hospital construction.	
External viability (EUR/lm)	Literature reference value: 800 EUR/lm
Distributive internal viability (EUR/lm)	Literature reference value: 600 EUR/lm
White sewer line connection (EUR/lm)	Literature reference value: 200 EUR/lm
Black sewer line connection (EUR/lm)	Literature reference value: 300 EUR/lm
Water connection (EUR/lm)	Literature reference value: 250 EUR/lm
Methane gas connection (EUR/lm)	Literature reference value: 150 EUR/lm
Electrical connection (EUR/lm)	Literature reference value: 100 EUR/lm
Telephone line connection (EUR/lm)	Literature reference value: 60 EUR/lm
Interior lighting work (EUR/lm)	Literature reference value: 350 EUR/lm
Pedestrian roads work (EUR/lm)	Literature reference value: 300 EUR/lm
Green areas (EUR/sqm)	Literature reference value: 40 EUR/sqm

**Table A3:** Building box mono-parametric construction costs.

In this section, the 3 different hospital complexity typologies (HUB, SPOKE, BASE) are reported, and it is required to insert a value in EUR/sqm and EUR/bed for their construction.	
High-complexity hospital (EUR/sqm)	Literature reference value: 2500 EUR/sqm
Medium-complexity hospital (EUR/sqm)	Literature reference value: 2100 EUR/sqm
Low-complexity hospital (EUR/sqm)	Literature reference value: 1800 EUR/sqm
High-complexity hospital (EUR/bed)	Literature reference value: 300,000 EUR/bed
Medium-complexity hospital (EUR/bed)	Literature reference value: 264,000 EUR/bed
Low-complexity hospital (EUR/bed)	Literature reference value: 252,000 EUR/bed

**Table A4:** Building box pluri-parametric construction costs.

In this section, it is required to insert a value in EUR/sqm for each category, according to own experience in hospital construction.	
Hospitalization (EUR/sqm)	Literature reference value: 2040 EUR/sqm
Intensive therapy (EUR/sqm)	Literature reference value: 2450 EUR/sqm
Operating rooms (EUR/sqm)	Literature reference value: 4050 EUR/sqm
Diagnosis and clinic (EUR/sqm)	Literature reference value: 1650 EUR/sqm
Reception (EUR/sqm)	Literature reference value: 1450 EUR/sqm
Support and logistic services (EUR/sqm)	Literature reference value: 1450 EUR/sqm
Administrative and management services (EUR/sqm)	Literature reference value: 1150 EUR/sqm
Connective (EUR/sqm)	Literature reference value: 1150 EUR/sqm
Technical rooms (EUR/sqm)	Literature reference value: 1150 EUR/sqm
Commerce and other functions (EUR/sqm)	Literature reference value: 1400 EUR/sqm
External parking (EUR/sqm)	Literature reference value: 150 EUR/sqm
Internal parking (EUR/sqm)	Literature reference value: 1100 EUR/sqm

**Table A5:** Technological units’ cost.

In this section, it is required to insert a value in EUR/sqm for the technological units by the hospital complexity typology, according to own experience in hospital construction	
High-complexity hospital (EUR/sqm)	Literature reference value: 367 EUR/sqm
Medium-complexity hospital (EUR/sqm)	Literature reference value: 354 EUR/sqm
Low-complexity hospital (EUR/sqm)	Literature reference value: 343 EUR/sqm

**Table A6:** Furniture cost.

In this section, it is required to insert a value in EUR/sqm for some sanitary furniture functions, according to own experience in hospital construction.	
Sanitary area furniture (EUR/sqm)	Literature reference value: 130 EUR/sqm
Services furniture (EUR/sqm)	Literature reference value: 120 EUR/sqm
Connective and technical room furniture (EUR/sqm)	Literature reference value: 30 EUR/sqm
